# Supplementary material for: Pooling sputum for Xpert MTB/RIF and Xpert Ultra testing during the Covid-19 pandemic in Lao People’s Democratic Republic
Source: PLOS Glob Public Health. 2022 Apr 4;2(4):e0000116. doi: 10.1371/journal.pgph.0000116 (PMC10022270; doi:10.1371/journal.pgph.0000116)
Supplement: Attachment — Submitted filename: Replies to reviewers comments_PCF_R2.docx [file pgph.0000116.s002.docx]

**Response to referees**

1. It does not look like your data has been uploaded as Supporting Information files yet. At this time, please upload your data.
The data have been uploaded as suggested.

2. Will the uploaded datasets qualify as your "minimal dataset" ?
Datasets qualify as "minimal dataset".

3. Please confirm that, once you upload your data, there will be no need to for the corresponding author to be contacted for data access.
Once data are uploaded, there is no need to inform the corresponding author for data access. However, a formal request should be made if data were to be used for another study.
