# Supplementary material for: Pooling sputum for Xpert MTB/RIF and Xpert Ultra testing during the Covid-19 pandemic in Lao People’s Democratic Republic
Source: PLOS Glob Public Health. 2022 Apr 4;2(4):e0000116. doi: 10.1371/journal.pgph.0000116 (PMC10022270; doi:10.1371/journal.pgph.0000116)
Supplement: Attachment — Submitted filename: Replies to reviewers comments_PCF_R3.docx [file pgph.0000116.s003.docx]

Reviewer’s comments

Line 92- please indicate the individuals as adults (as the study was done in adults)

Participants ranged from 1 to 98 years old. This is stated in the table and text. Although most participants were adults, children were not excluded, and we have not modified the text.

Line 139-why did you need ethical waiver because the usual way is to ask for informed consent for each individual during enrolment to the study?

Both ethics committees approved conducting the studies and waived the need for ethical approval. A “waiver” means that the committee considers that there is no need to obtain formal ethical approval from them. A waiver is only granted if the committee is satisfied and the study meets their requirements and this decision was based on:

•	Sputum specimens for clinical management arriving at the laboratory and tested individually under routine conditions. Test results being returned to healthcare workers for clinical management regardless of the pooling results.

•	The study only testing remnant specimens, which would have been discarded after routine testing. The pooled test results were only tested to document the performance of the test but were not used to guide TB treatment.

As these clarifications do not influence our results, we have not modified the text.

Line 145-46-the sentence mentioning that males are more likely to be xpert positive than females, does it have any explanation? and p<0.1 . I don’t think it is worth to mention unless there is some possible explanation or at least statistically significant( p value usually used is <0.05 to say statistically significant).

We had indicated in our previous response to referees that these differences are not statistically significant. Please see our previous reply, as pasted below:

***6. In Table 1, there are differences in demographic and Xpert results between the two groups (2020 and 2021 surveys). Have you tested whether these are significant? Was there any difference in the populations tested between years? Xpert Ultra is more sensitive, which would explain some of the differences. But would not explain why the proportion of male/female MTB detected would switch?***

*There was no statistically significant differences in the proportion of males and females recruited between 2020 and 2021 (p = 0.7), the proportion of males with MTB detected (72/491 (14.7%) vs 59/500 (11.8%), p = 0.18), nor the proportion of females detected (30/349 (8.6%) vs 41/340 (12.1%), p = 0.13). We have indicated in the text that these differences are not statistically significant. In general, TB is more frequent among males. We assumed the changes in the proportion of male/females detected was due to the surveys being conducted in different towns and this was a random event. Annual data reported by the country indicates incidence/prevalence is higher among males.*

We have denoted the p value “> 0.1”in the text to emphasize the differences were not statistically significant. If the editor wishes, they can be deleted, but this is usually the choice of the journal. As these clarifications do not influence our results, we have not modified the text further.

Line 157- Table 1 -please indicate the lowest age group in the range.

The age range is already included in row one, as pasted below (figures **1-98** and **1-96**). We have quoted these numbers in the text (see line 92 comment above).

| **Age (Mean (sd, range))** |  | 49 (19.4, 1-98) | 51 (19.6, 1-96) |
| --- | --- | --- | --- |
| **Age group (years)** | <35 | 237 (28.2) | 210 (25.0) |
|  | 35-54 | 238 (28.3) | 207 (24.6) |
|  | >=55 | 365 (43.5) | 423 (50.4) |

Line 268-270 The sentence “which may be attributed to the increased manipulation of samples resulting in an increased risk of contamination and labelling errors and these varying results may reflect the competency and dedicated time available of laboratory staff for sample processing.”
How did you reach to this argument? Was it stated by the studies as their limitation?

This is a comment in the discussion, where we aim to interpret the findings from other studies (not ours) and provide possible explanations why other studies may have reported lower specificity. We have not tested this hypothesis. However, if samples are manipulated in the lab, the risk of contamination increases, which is a well-established issue for other samples (e.g. increased contamination of gastric aspirates after the addition of buffers). As this is part of the discussion, we have not modified the text.
